# The effects of individual, occupational, and supportive factors on successful return to work using a structural equation model

**DOI:** 10.1186/s40557-015-0070-3

**Published:** 2015-08-28

**Authors:** Jongin Lee, Min Choi, Sung Hye Park, Hyoung-Ryoul Kim, Hye-Eun Lee

**Affiliations:** Department of Occupational and Environmental Medicine, College of Medicine, The Catholic University of Korea, Seoul, Korea; ᅟ, Korea Institute of Labor Safety and Health, Seoul, Korea

**Keywords:** Return to work, Structural equation model, Panel Study of Worker’s Compensation Insurance

## Abstract

**Objectives:**

We aimed to ascertain the relationship between several factors and successful return to work using a structural equation model.

**Methods:**

We used original data from the Panel Study of Worker’s Compensation Insurance, and defined four latent variables as occupational, individual, supportive, and successful return to work. Each latent variable was defined by its observed variables, including age, workplace size, and quality of the medical services. A theoretical model in which all latent variables had a relationship was suggested. After examining the model, we modified some pathways that were not significant or did not fit, and selected a final structural equation model that had the highest goodness of fit.

**Results:**

All three latent variables (occupational, individual, and supportive) showed statistically significant relationships with successful return to work. The occupational and supportive factors had relationships with each other, but there was no relationship between individual and the other factors. Nearly all observed variables had significance with their latent variables. The correlation coefficients from the latent variables to successful return to work were statistically significant and the indices for goodness of fit were satisfactory. In particular, four observed variables—handicap level, duration of convalescence, working duration, and support from the company—showed construct validities with high correlation coefficients.

**Conclusions:**

All factors that we examined are related to successful return to work. We should focus on the supportive factor the most because its variables are modifiable to promote a return to work by those injured in their workplace.

## Introduction

The purpose of industrial accident compensation insurance is not only to compensate workers who have work-related injuries in the workplace, but to promote the rehabilitation of accident victims and their return to society [1]. It is ideal for workers who have work-related injuries to return to their original work after sufficient treatment and recovery, but workers are unable to do so for various reasons.

Many studies have searched and analyzed factors related to returning to work. Factors such as age [2–5], sex (female) [3, 4, 6], level of education [4], economic status [6], severity of disease [2, 4], starting point of treatment [2], duration of convalescence [4, 5, 7], handicap level [5–7], working duration [2, 3, 5, 7], size of workplace [3, 5], occupational category [3, 5, 7], average wages [5], type of employment [7], and intervention for returning [8] have been described as important elements. Recent studies have shown that psychological elements—including self-efficacy, coping skill, disability acceptance, and depression—have a relationship with returning to work. Those studies have mainly identified the effect of various measurable factors on returning to original work to work more broadly as a dependent variable.

Meanwhile, the returning rate of workers suffering from work-related injuries (including self-employment) was low at 28.6 % in the 1990s in Korea [9]. However, in 2010, the rate increased to 64.1 % after the launch of various rehabilitation services, such as the establishment of a five-year plan for rehabilitation, installation of the responsible department for rehabilitation, and introduction of rehabilitation counselors [10]. While it is clear that the returning rate became higher, we should also consider whether such returns to work were successful.

Some studies show both the relationship between returning to work and other factors and also the subdivision of returning [5, 10], exploring the returning process [6], or tracing workers after returning [11]. Other studies have shown that returning to work would not be successful [12, 13] and unsuccessful returns made many injured workers change their jobs [11, 14]. If a worker had not returned to his or her original work, he or she could have had success via reemployment or self-employment. However, few studies have looked at these aspects.

The aim of this study is to establish a theoretical structural equation model by selecting variables known to relate to returning to work, and to explore the concept of a “successful return to work” and its particular variables. After ensuring the model is adequate, the final model will be determined and suggested.

## Materials and methods

### Study population

This study analyzed the Panel Study of Worker’s Compensation Insurance (PSWCI). The PSWCI is a panel study conducted by the Korea Workers’ Compensation and Welfare Service (KCOM) to follow up various indices among workers who have work-related injuries in the workplace and prepare the fundamentals of policies [15]. The study sampled 2000 workers from 82,493 who had finished their convalescence in 2012. The workers are to be followed for five years, and the data from the first year of the study was used in this study. The 2000 workers were sampled by stratification according to their handicap level, location, and experience of rehabilitation services from the KCOM. However, sex, age, and duration of convalescence were not stratified although they are also important; these indices were sampled systematically according to the number of each sample because it is not easy to stratify with so many indices. But by systematical sampling with these three variables, the design of PSWCI reflected the weight of these indices. The sample was weighted, allowing the 2000 workers to represent all 82,493 workers.

### Structural equation modeling

This is a multivariate analysis technique used to ascertain causality among variables, analyzing the relationships among variables, and clarifying the structural relationship [16]. We selected the observed variables that can be measured using the PSWCI data by referring to previous studies. R version 3.1.1 was used in the analysis. Because the PSCWI is a panel data, all variables were calculated with weighted value.

### Definition of observed variables

Observed variables were defined as below (Tables 2 and 3); Age, handicap level, and duration of convalescence: The range of each variable was as follows: 1 (under 20) to 5 (at least 60 or more), 1 (Level 1 to 3) to 6 (no handicap level), and 1 (3 months or under) to 6 (more than 2 years), respectively.

Size of workplace and working duration: The range of each variable was as follows: 1 (fewer than 5 people) to 7 (at least 1000 people or more), and 1 (under 1 month) to 14 (at least 20 years or more), respectively. The variables were modified to conform to a maximum score of 5.

Average wage: This was a subjective question, from 50,000 Korean Won (least) to 9 million Korean Won (most). The score was given to each stratum, divided by 2 million Korean Won, from 1 (under two million Korean Won) to 5 (at least eight million Korean Won or more).

Quality of medical services: The score was formed by combining questionnaires assessing medical services; workers were asked about doctor treatment plans, periodic assessment for recovery, and appropriate treatment duration. All questions were Yes/No, and the score ranged from 0 (not satisfactory at all) to 3 (all satisfactory services).

Interest of attending physician for returning and opinion profile service from the KCOM: KCOM provides services of opinion profile (a doctor’s assessment sheet whether the injured worker could work or not). The score from the original data were marked from 1 (minimum) if the worker had full satisfaction to 5 (maximum) if there was no satisfaction at all. Considering the direction of our model, the score was modified to have a maximum of 5 if the worker had full satisfaction. If the worker did not experience consultation from an attending physician nor accepted an opinion profile service, a score of 0 was given.

Support from company, general job satisfaction, and daily-life satisfaction: Similar to the variables above, the scores for these factors were modified to have a maximum of 5 points if the worker felt full satisfaction. The daily life satisfaction became a single question, and the means of 6 items (income, leisure, accommodation, family, relatives, and social acquaintance) were used.

Personal job satisfaction and work-environmental satisfaction: The PSWCI asked different questions according to the worker’s current occupational state: return to original work, re-employment, self-employment, unpaid family worker, loss of job, or not economically active state. Common questionnaires for all states were used for the variables. The maximum satisfaction score was marked as 5; if the injured worker was not employed, a score of 0 was given.

Self-efficacy and self-esteem: The Self-Efficacy Scale (SES) by Sherer and colleagues [17] and the self-esteem scale by Rosenberg [18] were investigated in the original data. The average scores of each scale for the original data were used in the analysis. The maximum scores of the SES and the Rosenberg scale were 4 and 5, respectively. Each Korean version is translated, adapted and tested in its reliability and validity by Jon [19] and Hong [20].

### Definition of latent variables

After the observed variables were selected, these were categorized into four latent variables: individual factor, occupational factor, supportive factor, and successful return to work. Each latent variable and its observed variables are demonstrated in Table 1.

**Table 1:** Latent variables and their observed variables

Latent variables	Observed variables
Individual	Age, Handicap level, Duration of convalescence
	Self-efficacy, Self-esteem
Occupational	Size of workplace, Working duration, Average wage
Supportive	Quality of medical services, Interest of attending physician for returning to work, Opinion profile service from KCOM, Support from company
Successful return to work	Personal job satisfaction, Work environment satisfaction, General job satisfaction, Daily life satisfaction

We defined a term ‘successful return to work’ as a latent variable affected by satisfaction scores in four aspects – personal job satisfaction, work environment satisfaction, general job satisfaction, and daily life satisfaction.

### Theoretical structural equation model

**Fig. 1:**
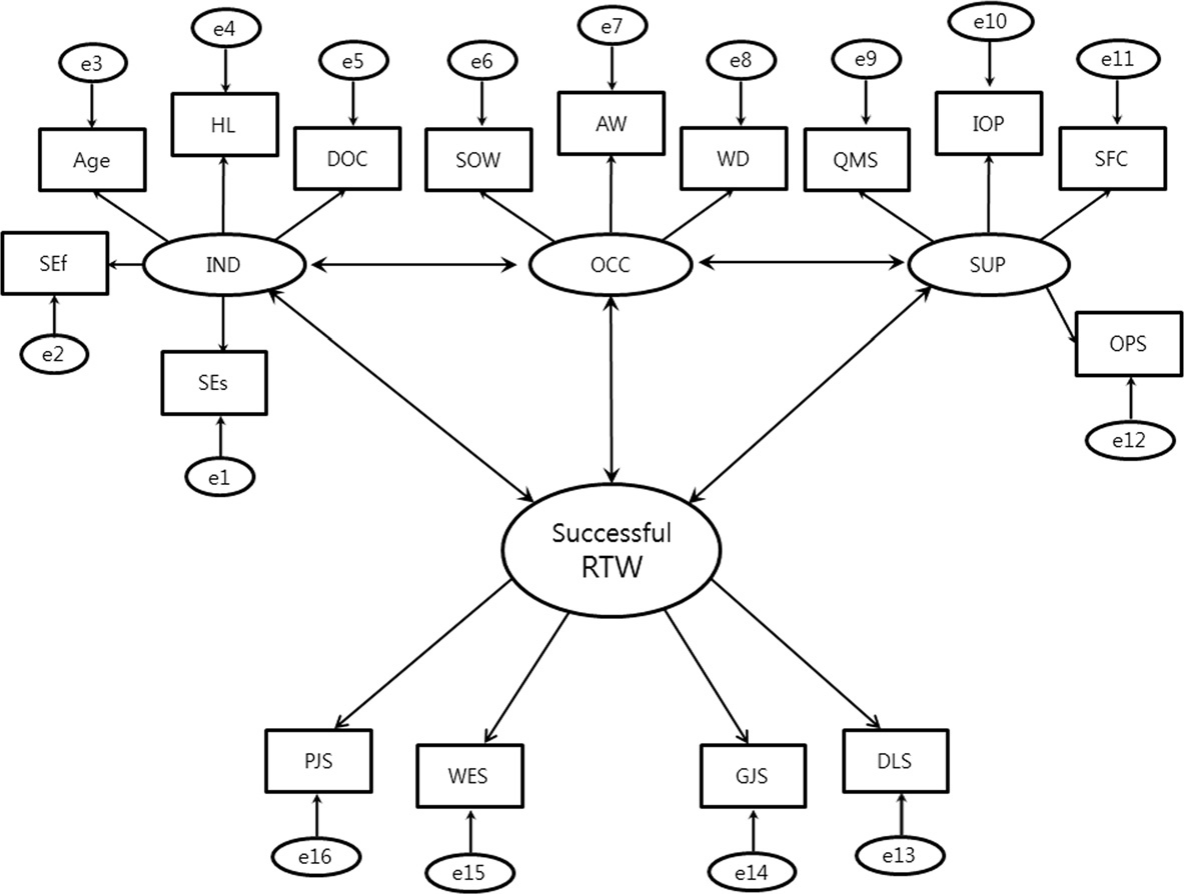
Theoretical structural equation model. *Relationship between the individual and supportive factors is not described due to lack of space. **Abbreviations: IND: Individual factor, OCC: Occupational factor, SUP: Supportive factor, RTW: Return to work, SEf: Self-efficacy, SEs: Self-esteem, HL: Handicap level, DOC: Duration of convalescence, SOW: Size of workplace, AW: Average wage, WD: Working duration, QMS: Quality of medical services, IOP: Interest of attending physician for returning to work, SFC: Support from the company, OPS: Opinion profile service from KCOM, PJS: Personal job satisfaction, WES: Work environment satisfaction, GJS: General job satisfaction, DLS: Daily life satisfaction

We hypothesized that the individual, occupational, and supportive factors could have an effect on successful return to work. However, the causal relationship was not definite because the survey is cross-sectional, and the relationships between the latent variables were assumed to be correlations and not causal. Other relationships among the factors could exist rather than with successful return to work. The theoretical model is shown in Fig. 1 (the relationship between the individual and supportive factors is not described due to insufficient space). The correlation coefficients were calculated with standardization. Statistical significance was considered met if *p* < 0.05.

In our primary theoretical model, all relationships between individual, occupational, and supportive factors with successful return to work showed statistical significance, whereas relationships between individual and occupational factors and between individual and supportive factors showed low correlation coefficients and were not significant. Therefore, these two relationships were deleted in the final model.

## Results

### Participants’ characteristics

The demographic characteristics are shown in Table 2. Most were male (84.3 %) and in their 50s (35.2 %). The most frequent handicap level was 10th to 12th grade (40.7 %). They had mostly worked for a relatively short duration (under 1 month, 32.6 %), and had recovered for predominantly 3 to 6 months (41.3 %). The number of employed workers was 70.6 %, which was more than in the previous studies.

**Table 2:** Socio-demographic and work-related characteristics of PSCWI participants

Latent variables	Characteristics	Classification	*N* = 2000	Percent
	Sex	Male	1686	84.3
		Female	314	15.7
Individual	Age	Under 20s	118	5.9
		30s	295	14.8
		40s	522	26.1
		50s	705	35.3
		Over 60s	360	18.0
	Handicap Level	1st to 9th	265	13.3
		10th to 14th	1385	69.3
		No handicap	350	17.5
	Duration of Convalescence	No more than six months	1148	57.4
		No more than one year	647	32.4
		Over one year	205	10.3
Occupational	Size of workplace	<30	1478	73.9
	(people)	30–299	437	21.9
		300–999	46	2.3
		≥1000	39	1.9
	Working Duration	Under one year	1312	65.7
		At least one year or above	688	34.3
	Wage (Won/monthly)	<2,000,000	819	40.9
		2,000,000–4,000,000	1036	51.8
		4,000,000–6,000,000	133	6.7
		6,000,000–8,000,000	10	0.5
		≥8,000,000	2	0.1

Table 3 shows observed variables that are appraised by mean and deviation. The means were lowest in interest of attending physician and opinion profile service from KCOM.

**Table 3:** Observed variables with weighted mean and standard deviation

Latent variables	Observed variables	Weighted mean
Individual	Self-efficacy	4.17 ± 0.51
	Self-esteem	2.87 ± 0.39
Supportive	Quality of medical services,	3.98 ± 1.25
	Interest of attending physician for returning to work	0.94 ± 1.69
	Opinion profile service from KCOM	0.27 ± 0.99
	Support from company	2.82 ± 0.91
Successful	Personal job satisfaction	2.73 ± 1.62
return to work	Work environment satisfaction	2.54 ± 1.50
	General job satisfaction,	2.56 ± 1.54
	Daily life satisfaction	3.30 ± 0.52

### Confirmative structural modeling

**Fig. 2:**
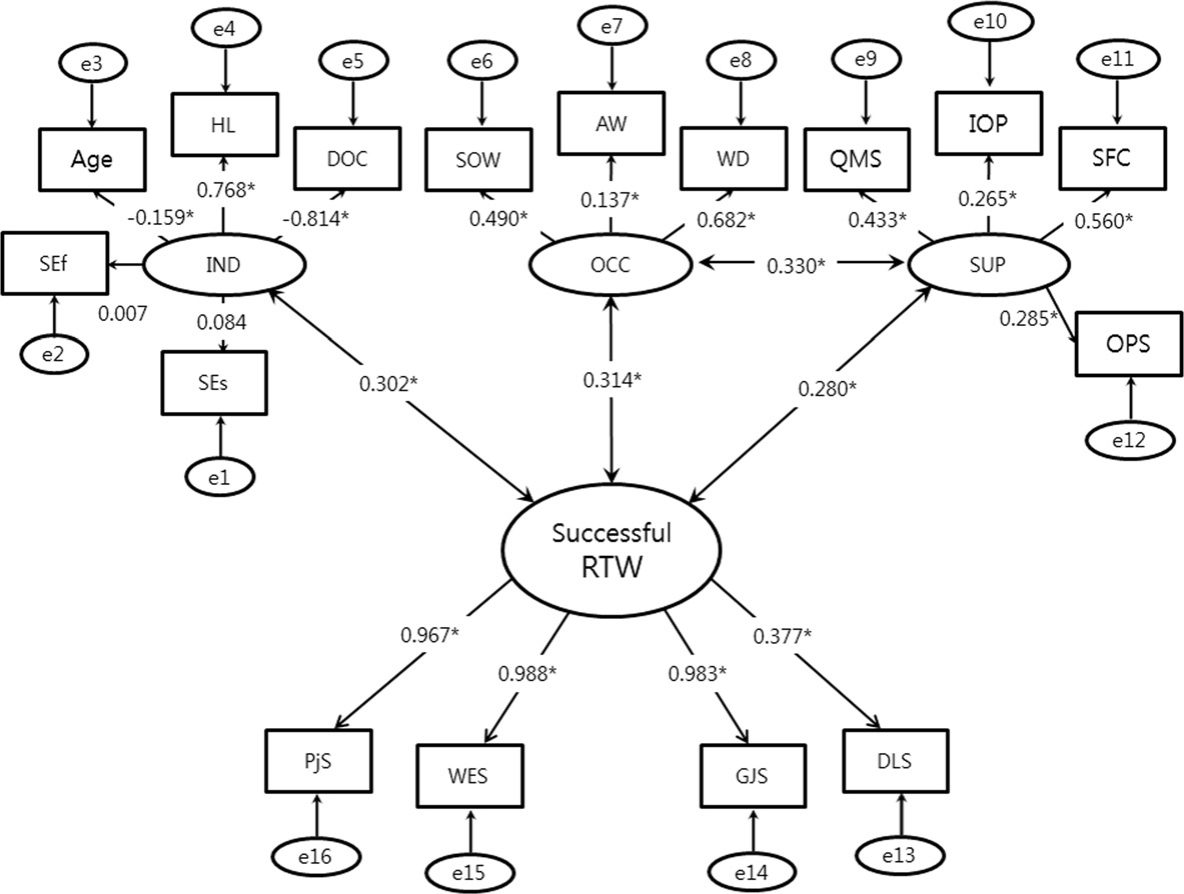
Final structural equation model. *Abbreviations: IND: Individual factor, OCC: Occupational factor, SUP: Supportive factor, RTW: Return to work, SEf: Self-efficacy, SEs: Self-esteem, HL: Handicap level, DOC: Duration of convalescence, SOW: Size of workplace, AW: Average wage, WD: Working duration, QMS: Quality of medical services, IOP: Interest of attending physician for returning to work, SFC: Support from the company, OPS: Opinion profile service from KCOM, PJS: Personal job satisfaction, WES: Work environment satisfaction, GJS: General job satisfaction, DLS: Daily life satisfaction

We selected the final model, which outlined that all the individual, occupational, and supportive factors had relationships with successful return to work (correlation coefficients were 0.302, 0.314, and 0.280, respectively) and there was a relationship between occupational and supportive factors (Fig. 2).

Regarding relationships between each latent variable and its observed variables, the individual factor was explained well by the handicap level and the duration of convalescence but not by SES and Rosenberg’s scale, which were not significant and were not highly correlated. Therefore, after the two scales were classified into a new factor, “internal,” we tried to analyze the model again. However, the new model did not yield any result because it did not converge. The workplace size and working duration were important for the occupational factor, and support from the company and quality of medical services were important for the supportive factor. All three observed variables for successful return to work were significant, despite the coefficient correlation of daily life satisfaction being lower than the others.

The chi-square fitness of the final model was 837.89 (df 100); *p* < 0.001 meant that it was non-significant. However, all the other fitness indices were favorable: adjusted goodness of fit index (AGFI) was 0.998, root mean squared error of approximation (RMSEA) was 0.061, comparative fit index was 0.947, and Tucker-Lewis index was 0.936. If RMSEA is under 0.08, the model is favorable [21].

### Gender difference

**Fig. 3:**
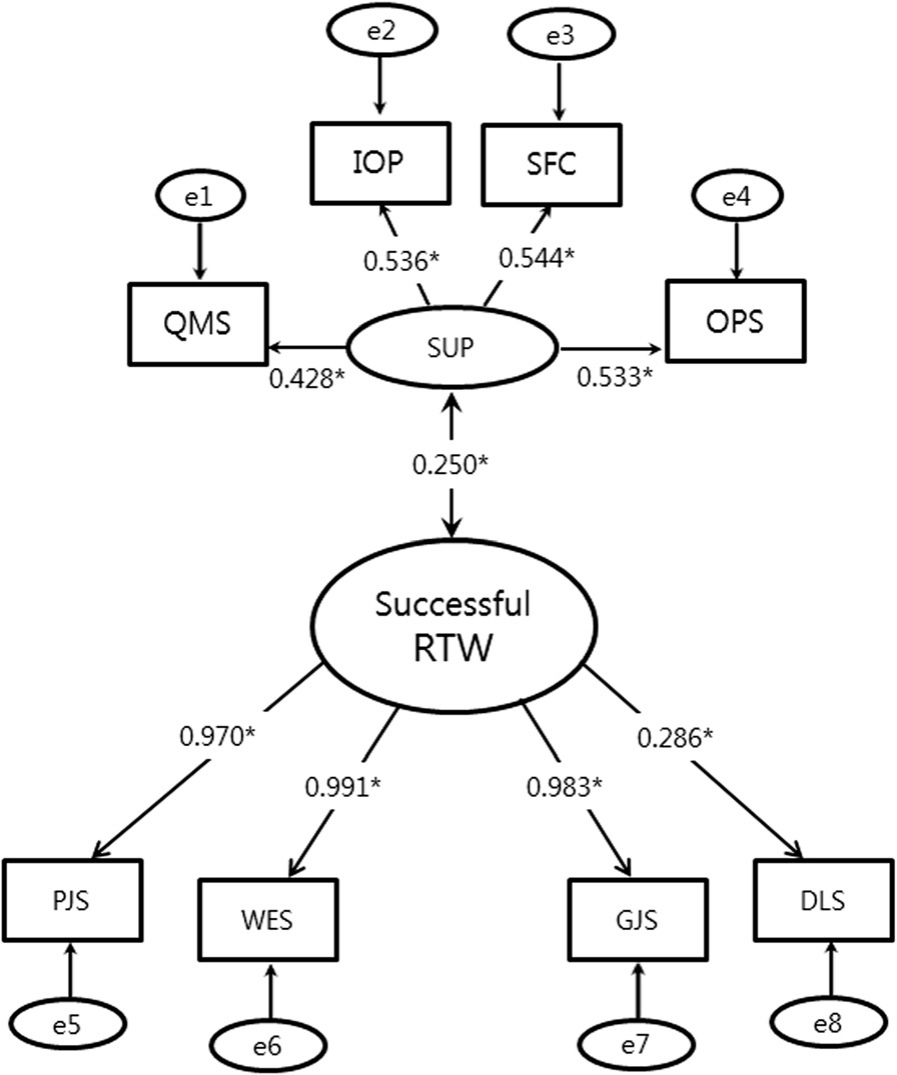
The sub-analysis model for women. *Abbreviations: SUP: Supportive factor, RTW: Return to work, QMS: Quality of medical services, IOP: Interest of attending physician for returning to work, SFC: Support from the company, OPS: Opinion profile service from KCOM, PJS: Personal job satisfaction, WES: Work environment satisfaction, GJS: General job satisfaction, DLS: Daily life satisfaction

Because previous studies have shown a difference between each gender, the sub-analysis was conducted after dividing the two genders. Whereas the overall direction of the model did not change in males, all observed variables explaining the individual and occupational factors lost their significance in females. Therefore, the model in which the individual and occupational factors were deleted was confirmed as a final model for females, and there was a significant relationship between the supportive factor and successful return to work (Fig. 3). The fitness indices for the two models were favorable with AGFI, RMSEA, CFI, and TLI, except for chi-square fitness.

## Discussion

In this study, we defined successful return to work and selected the variables that have an effect on this to analyze relationships among the variables using structural equation modeling.

Three latent variables expected to be related to successful return to work showed positive relationships with statistical significance. However, all correlation coefficients were below 0.5. All fitness indices were favorable except chi-square fitness. This is because the chi-square fitness is not adjustable if the sample size is large [16]. We judged that our model fit well because all other fitness indices were favorable.

Each observed variable had a significant relationship with its latent variable. The direction of the variables corresponded with the hypothesis. All variables that had positive relationships with their latent variables had desirable characteristics with their increasing scores. Only age and duration of convalescence had negative relationships with the individual factor. Variables that had high explanatory power were handicap level and duration of convalescence for the individual factor, working duration for the occupational factor, and support from the company for the supportive factor. It was noted in many previous studies that these variables affected returning to work; this study showed that they were not just important to returning to work itself, but to successfully returning to work, and, importantly, the satisfaction of the injured workers.

Among the observed variables for successful return to work, measurements for satisfaction of personal job, work environment, and general job showed very high correlations with their latent variable. Daily life satisfaction also had a significant relationship, although its explanatory power was relatively lower. Therefore, it was desirable to use these satisfaction measurements in defining a successful return to work.

With stratification by gender, the male model was similar to the whole, while all variables for the individual and occupational factors did not explain their latent variable in the female model. There could be two possibilities for this: 1) the proportion of women is lower than that of men, and 2) the gender factor had greater influence on successful return to work compared to any other factors, so the effect of the other factors was diluted.

Self-efficacy and self-esteem could not explain the individual factor sufficiently. Although we could not analyze their effects further because the model did not converge with their separation to a new latent variable, more thorough analysis is needed because recent studies have shown that internal factors such as these had meditative effects on returning to work [6, 22] or affective aspects of returned workers [23].

The points that are modifiable by a policy are the variables for the supportive factor. The most effective one was support from the company. The quality of the medical services was also important. The interest of the attending physician for returning to work and the opinion profile service from the KCOM had lower correlation coefficients, but they were still statistically significant. The reason why these two variables had lower explanatory power may be that only a few experienced these services. A study had highlighted the importance of a systematic approach to facilitate early return to work [24]; therefore, the role of attending physicians in the system should be emphasized. The average values of the two services in the PSWCI survey were 0.94 ± 1.69 and 0.27 ± 0.99 out of 5.00, respectively. If there is a policy that focuses on these variables for the supportive factor, it is expected that the effect will be optimal for a successful return to work.

However, current system of worker’s compensation does not support injured workers sufficiently. The workers often experience intimidating aspects of the compensation system [25]. They are neglected. They are not treated soundly. Sometimes they are forced to return to work even though they are not cured fully. In contrast, KCOM prepares a lot of novel services such as opinion profile service and support for rehabilitation. But it should be considered whether the service is delivered effectively to the workers [26].

This study has some limitations. We used the first year data from the PSWCI so the study design was cross-sectional. Therefore, the relationship we showed cannot be causal. We could not analyze some factors that were known to have effects on the return to work in previous studies but that were not surveyed in the PSWCI. The effect by gender was not clarified because analysis for dichotomous variables is not desirable in structural equation modeling. Instead, we analyzed the gender difference by stratification—many effects disappeared in women, which may be due to the effect of gender being greater than any other factor.

Accident- or disease-related factors are important in terms of returning to work. Although returning to work will be different according to the cause of the industrial injury or disease, medical information, such as diagnosis, was not surveyed in the PSWCI. No other indicator suggested the severity of the injury except duration of convalescence and handicap level. Causes of convalescence or disease classifications should be inquired at least to assess related factors that have impact on returning to work in further investigations of PSWCI in order to control the effect of severity of diseases or injuries.

Defining a novel term ‘successful return to work’ might be controversial. A previous study focused on the importance of employment status and life satisfaction simultaneously on occupationally injured people [27]. But it is not reasonable to combine merely two kinds of scores – occupation and daily life. But by using structural equation modeling, we could combine these aspects into a unified concept that is the strength of this study. We also considered weighted values that allowed the representativeness of all injured workers in 2012. Further studies are needed to find other factors and paths to returning to work, and clarity will increase annually with the PSWCI.

## Conclusions

In conclusion, all factors—individual, occupational, and supportive factors—affected successful return to work. Intervention for the particularly modifiable factors—in other words, the supportive factor in this study—could be helpful for injured workers to satisfactorily return to work.
